# Convergent structure and function of mycelial galleries in two unrelated Neotropical plant-ants

**DOI:** 10.1007/s00040-017-0554-y

**Published:** 2017-03-11

**Authors:** V. E. Mayer, J. Lauth, J. Orivel

**Affiliations:** 1 Department of Botany and Biodiversity Research, Division of Structural and Functional Botany, University of Vienna, Rennweg 14, 1030 Vienna, Austria; 2 CNRS, UMR Ecologie des Forêts de Guyane, AgroParisTech, CIRAD, INRA, Université de Guyane, Université des Antilles, Campus Agronomique, BP316, 97379 Kourou Cedex, France

**Keywords:** Ant–plant interaction, Ambush behaviour, *Allomerus decemarticulatus*, *Azteca brevis*, Chaetothyriales, *Hirtella physophora*, Runway galleries, *Tetrathylacium macrophyllum*

## Abstract

The construction process and use of galleries by *Azteca brevis* (Myrmicinae: Dolichoderinae) inhabiting *Tetrathylacium macrophyllum* (Salicaceae) were compared with *Allomerus decemarticulatus* (Myrmicinae: Solenopsidini) galleries on *Hirtella physophora* (Chrysobalanaceae). Though the two ant species are phylogenetically distant, the gallery structure seems to be surprisingly similar and structurally convergent: both are pierced with numerous holes and both ant species use Chaetothyrialean fungi to strengthen the gallery walls. *Al. decemarticulatus* is known to use the galleries for prey capture and whether this is also the case for *Az. brevis* was tested in field experiments. We placed *Atta* workers as potential prey/threat on the galleries and recorded the behaviour of both ant species. We found considerable behavioural differences between them: *Al. decemarticulatus* was quicker and more efficient at capture than was *Az. brevis*. While most *Atta* workers were captured after the first 5 min by *Al. decemarticulatus*, significantly fewer were captured by *Az. brevis* even after 20 min. Moreover, the captured *Atta* were sometimes simply discarded and not taken to the nest by *Az. brevis*. As a consequence, the major function of the galleries built by *Az. brevis* may, therefore, be defense against intruders in contrast to *Al. decemarticulatus* which uses them mainly for prey capture. This may be due to a higher need for protein in *Al. decemarticulatus* compared to coccid-raising *Az. brevis*.

**Electronic supplementary material:**

The online version of this article (doi:10.1007/s00040-017-0554-y) contains supplementary material, which is available to authorized users.

## Introduction

The construction of independent nests, galleries, shelters, and inner walls that divide nests into separate cavities is a common feature in ants, particularly in arboreal species, built to provide colonies and their resources protection from climatic stress, parasites, and predators (Anderson and McShea 2001). Most species use carton made from plant fibres and debris or soil particles as building material. In the Neotropics, species from the genus *Allomerus* (Formicidae: Myrmicinae), as well as a species from the genus *Azteca* (Formicidae: Dolichoderinae), *Az. brevis* Forel, are known to build particular galleries on their host-plant stems; these galleries are pierced with holes (Dejean et al. 2004, 2005; Mayer and Voglmayr 2009). Moreover, the galleries of these ant species are reinforced with a dense net of fungal hyphae, which have been identified as members of the ascomycete order Chaetothyriales (Mayer and Voglmayr 2009; Nepel et al. 2014; Ruiz-González et al. 2011). Such apparent similarities in the galleries built by ant species from phylogenetically distant genera belonging to different subfamilies raise the question whether the construction process and the function of the holes are also similar. For *Allomerus* spp., it is known that the galleries are used for prey capture with ant workers waiting inside the galleries with their heads just under the holes and mandibles wide open. Prey that walk over or settle on the gallery are grasped by their legs, antennae, or wings, stretched against the gallery, killed, and taken to the nest (Dejean et al. 2005).

However, it has never before been investigated how *Az. brevis* uses its galleries, and whether these galleries have a function in prey capture analogous to those built by *Allomerus*. Therefore, we investigated the similarities and differences in the behaviour of the two ant species. We compared (1) the construction process and (2) experimentally tested whether the holes in the galleries built by *Az. brevis* have a prey capture function analogous to those built by *Al. decemarticulatus*.

## Materials and methods

### Species and study sites

*Azteca brevis* (Forel 1899) (Dolichoderinae) was studied in the *Parque Nacional Piedras Blancas* (Costa Rica) on *Tetrathylacium macrophyllum* growing near the La Gamba Biological Research Station (08°42′46″N, 83°12′90″W) (*n* = 4), along Rio Sardinal (08°43′48″N 83°13′28″W; *n* = 3) and along Rio Bolsa (08°41′06″N 83°10′35″W; *n* = 2) in a lowland primary rainforest characterised by high annual rainfall (>5000 mm). *Az. brevis* is a red-brown ant, ca. 4-mm-long, known only from the Pacific side of Costa Rica, mostly in the wet forests of the southern Pacific lowlands (Longino 2007a). The colonies are polydomous with cauline nests in the live stems of trees (Supplementary Fig. S1). All of the nesting chambers inside the stems are connected externally by an extensive system of galleries made of a characteristically black carton with small, circular holes. *Az. brevis* attends large populations of coccoid Hemiptera of the genus *Cryptostigma* (P. Gullan, pers. com.) inside the stems and in the runway tunnels (Supplementary Fig. S1C). Colonies have been found on *T. macrophyllum* (Salicaceae), a tree with an average height of about 8 m (but reaching a maximum height of 15–20 m) that grows on the Pacific slopes of Central and South America from southern Costa Rica to Ecuador with its centre of distribution in the Colombian Chocó (Supplementary Fig. S1A). It grows preferentially on steep slopes near rivers and streams in the primary forest (Janzen 1983). The domatia are located on the stems and develop from the partial degeneration of the pith. In the study area, the plant can be colonised by various ant species (Tennant 1989), but *Az. brevis* is the only one which actively enlarges the domatia by excavating the remaining pith (Schmidt 2001).

*Allomerus decemarticulatus* was studied in the area of *Petit Saut*, Sinnamary, French Guiana (05°03′30″ N, 52°58′34″W) in a lowland primary rainforest. *Al. decemarticulatus* (Mayr 1878) (Myrmicinae) are minute (1.5–1.8 mm), tawny-coloured ants that live strictly on ant-plants. In the study area, *Al. decemarticulatus* is the main obligatory inhabitant of *Hirtella physophora* (Chrysobalanaceae) with a single mature colony per plant; this ant species has never been found associated with other myrmecophytic species (Grangier et al. 2009). *H. physophora* is a long-lived understory treelet that has leaves with a pair of domatia at the base of each lamina (Leroy et al. 2008; Orivel et al. 2011). The species occurs mostly in patches on the upper slopes of hillsides (Solano et al. 2003).

### Investigation of the galleries’ structure and construction process

Inner gallery width, height, and hole diameter were measured with calipers. The measurements were taken from 2-cm-long pieces of carton cut from the galleries of four different colonies of *Az. brevis* and two colonies of *Al. decemarticulatus*. Head width, head length (including the mandibles), and the total body length of *Al. decemarticulatus* (*n* = 10) and *Az. brevis* (*n* = 5) workers were also measured.

The construction process of the carton galleries was studied for 15 different *Az. brevis* colonies during both the dry and the rainy seasons. We removed 10-cm-long sections of the carton galleries and recorded the reconstruction of the galleries by taking a photograph twice daily during 2 weeks. If recently developed branches of the host tree *T. macrophyllum* were easily accessible, we also monitored the continuation of the galleries on these new shoots.

Similar observations were made on ten *Al. decemarticulatus* colonies during the rainy season. The *Al. decemarticulatus* galleries were removed from the plant and gallery reconstruction was observed during 2 weeks. We documented the construction by photographing the galleries every day.

### Testing the function of the galleries of *Az. brevis* and *Al. decemarticulatus*

To test the role of the *Az. brevis* galleries and compare it with that of *Al. decemarticulatus* galleries, leaf-cutter ants (e.g. *Atta colombica* or *At. cephalotes*) were taken from nearby nests and placed on the galleries to examine the ants’ behaviour towards potential invaders or prey. *Atta* individuals are among the size range of prey that *Al. decemarticulatus* can capture (Dejean et al. 2005). Since *Az. brevis* workers are even larger than *Al. decemarticulatus* workers, we hypothesised that they should also be able to capture *Atta* ants.

The experiment was conducted on ten *Al. decemarticulatus*/*H. physophora* and nine *Az. brevis*/*T. macrophyllum* associations. For each plant, three separate and easily accessible branches were selected. One *Atta* individual was carefully placed with tweezers on an *Allomerus*-occupied *H. physophora* branch as well as on the upper side of an *Azteca*-occupied *T. macrophyllum* branch. The fate of the *Atta* ants was recorded after four time intervals (5, 10, 20, 30 min) and classified into one of three different categories: (1) remained free, (2) captured by the inhabiting ants, or (3) escaped from the tree by either dropping or running away. In a control experiment, the carton galleries of three branches from the same trees as before were destroyed and the behaviour of *Az. brevis* towards the leaf-cutter ants monitored. *Az. brevis* did not attack *Atta* individuals that were not on the carton galleries.

Through a Mann–Whitney *U* test for independent groups with non-parametric data, we tested the null hypothesis that the capture efficiency of *Al. decemarticulatus* and *Az. brevis* is the same for each time interval. The test was performed with IBM SPSS Statistics 23.

## Results

### Gallery structure, construction material, and process

**Fig. 1:**
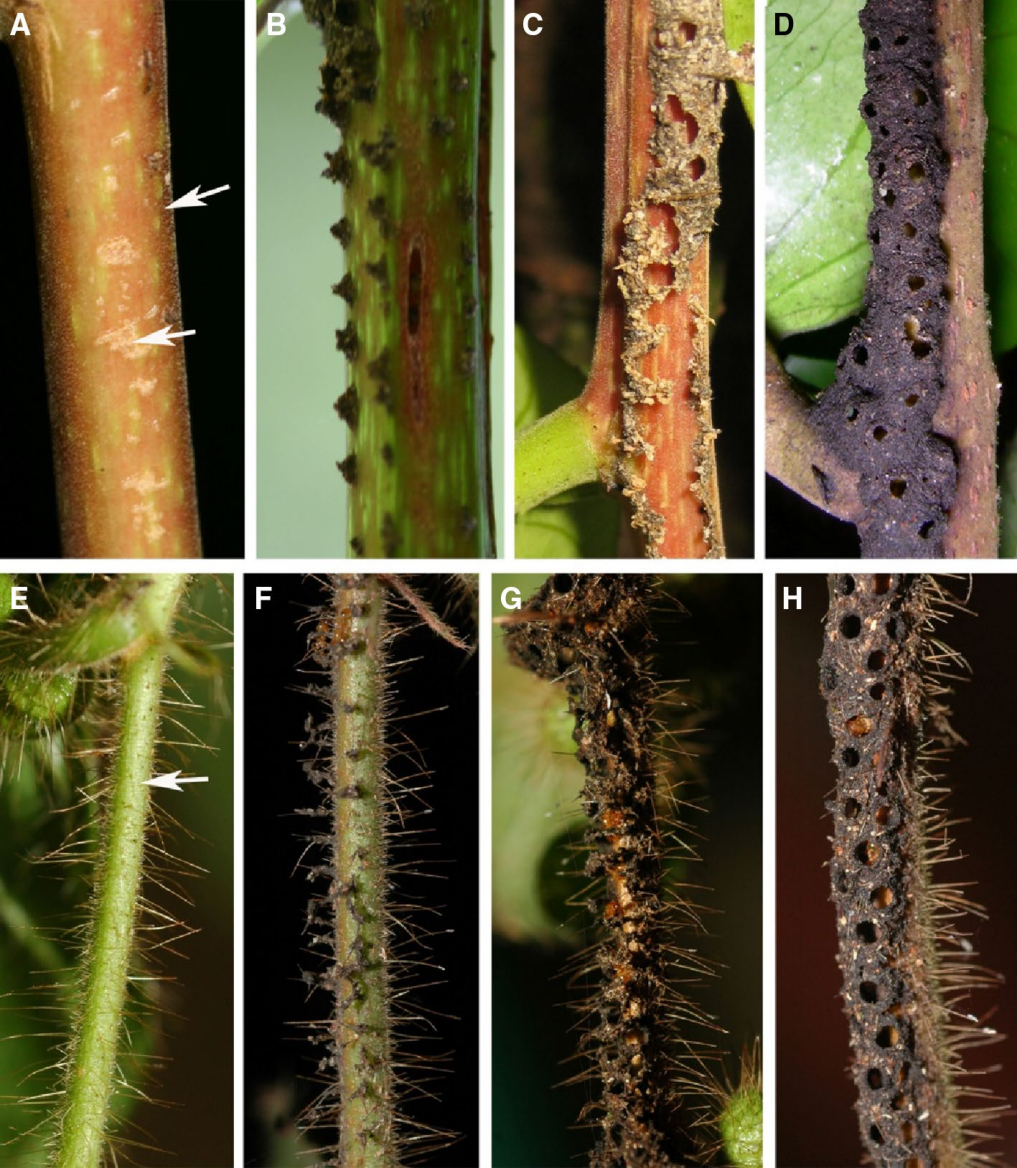
Construction of the carton galleries by *Az. brevis* (**a**–**d**) and *Al. decemarticulatus* (**e**–**h**). Both ant species start by clearing a path; *Az. brevis* also scrapes tissue from the bark (*arrows*) (**a, e**). *Az. brevis* uses chewed plant tissue to build rows of parallel pillars (**b**) and fills the space between (**c**). Holes are left during the construction process (**d**). *Al. decemarticulatus* clears a path by cutting the trichomes (**e**), which are then used to form the vault of the galleries (**f**–**g**). As for *Az. brevis*, the holes are not pierced after construction but left open during the process (**h**). The construction process was observed in the field in Costa Rica (*Az. brevis*) and in French Guiana (*Al. decemarticulatus*)

**Fig. 2:**
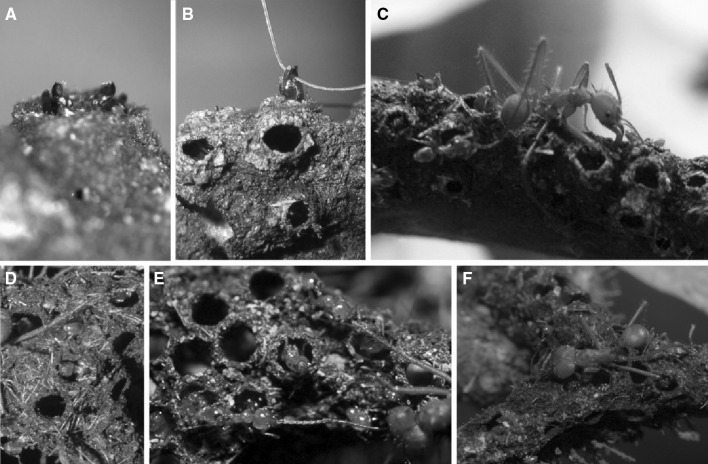
Function of *Az. brevis* and *Al. decemarticulatus* galleries. If the *Az. brevis* colony is disturbed, the workers take up positions beneath the holes and keep their mandibles wide open (**a**), grasping any item possible (**b**). In the latter photo, it is a strand of the first author’s hair, but usually *Az. brevis* ants grasp the legs or antennae of other arthropods (**c**). The prey is immobilised and killed. *Al. decemarticulatus* workers guard the holes throughout the day (**d**), and the appendices of the prey walking on the galleries are immediately caught and the prey immobilised (**e, f**)

For both species, the galleries were similar; i.e., an arched tunnel 2-mm-high and with an inner width of 3–4 mm. For *Azteca—*as well as *Allomerus*—built galleries, wall thickness was 0.2 mm, and the walls were perforated with ± evenly distributed circular holes which were ca. 1-mm-wide for both species, and separated by a distance of 1.3–6.0 mm for *Az. brevis* galleries and 2.0–4.5 mm for *Al. decemarticulatus* galleries (Fig. 1d, h; supplementary Table 1). The diameter of the holes was slightly larger than the workers’ heads with on average 0.96 mm for *Az. brevis* galleries and 0.55 mm for *Al. decemarticulatus* galleries (supplementary Table 1), allowing the ants to enter and exit the galleries. For *Az. brevis*, the holes were lined with a bulky ring (Figs. 1d, 2a, b). The galleries usually covered the underside of the branches (Fig. S1, supplementary material), including the entrances to the domatia. The holes were not pierced into completed galleries, but were made during the galleries’ construction (Fig. 1d, h).

For both ant species, new galleries were built almost exclusively with plant material. However, the material itself and the way they used it differed considerably. *Az. brevis* formed pellets from pieces of bark or epiphylls taken from the surface of their host-plant’s branches as well as pithy tissue obtained from excavating the branches (Fig. 1a) with which they first built two parallel rows of pillars (Fig. 1b), then a wall between each pair of adjacent pillars, and finally an arch to form the vault of the galleries (Fig. 1c). During the dry season, the construction progress was slow and even stopped after several days without rain, whereas, during the rainy season, up to 4 cm of gallery were built in a day.

*Allomerus decemarticulatus* workers did not use bark, epiphylls, or pithy tissue. Instead, they cut trichomes from the surface of the internodes located between the leaf pouches (Fig. 1e) and assembled them together to form the vault of the gallery. Adjacent, uncut trichomes served as pillars, and pellets made from tissue scratched off the inner domatia walls were pasted along the trichomes in the gallery (Fig. 1e–g) (Dejean et al. 2005; Ruiz-González et al. 2011). Then, the ants pasted pellets of hyphae from older gallery sections onto the plant material on newly built parts. The fungus then colonised the galleries, reinforcing them.

### Function of the galleries

**Fig. 3:**
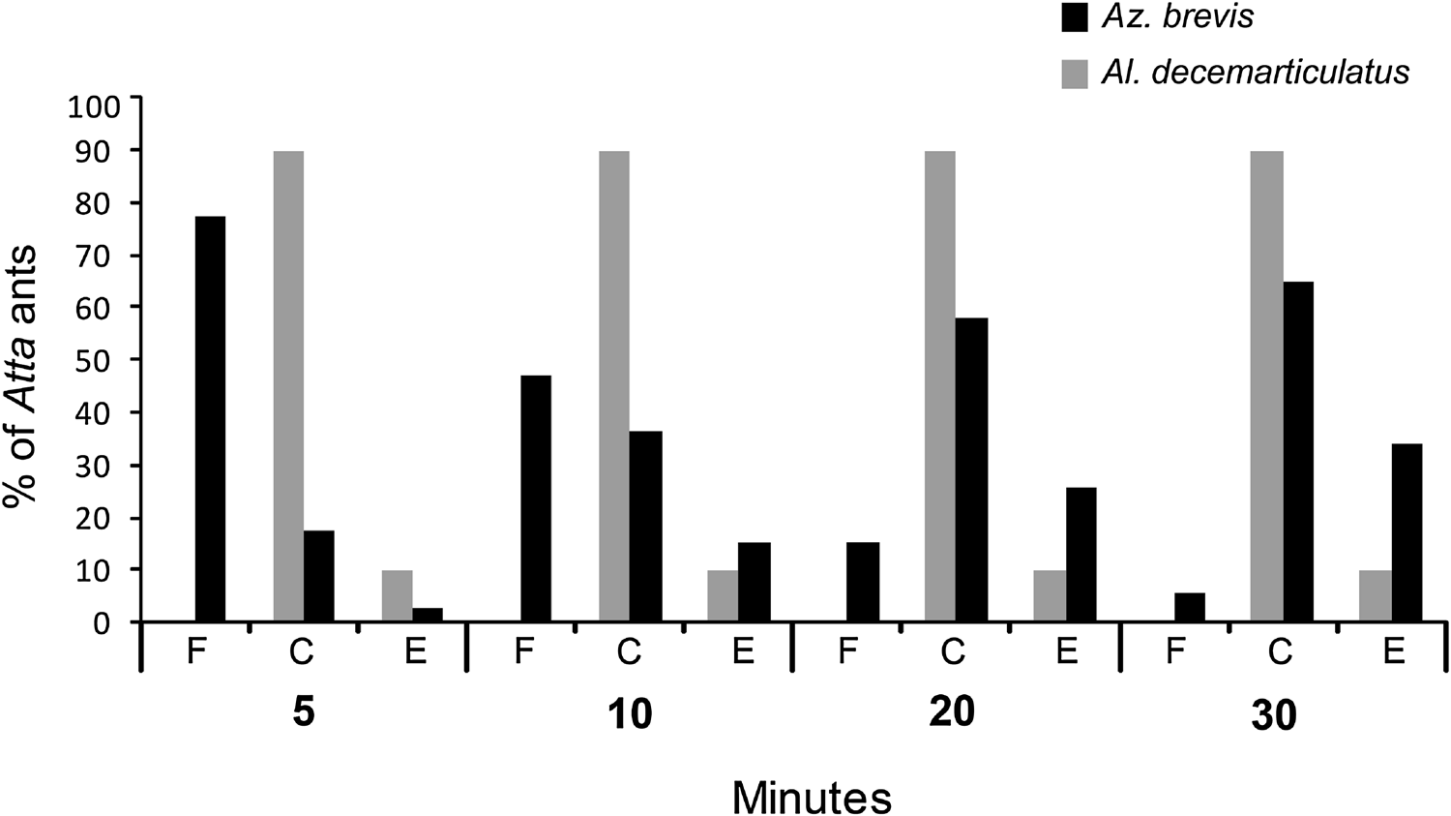
Experiment to test whether the galleries serve as traps for *Az. brevis* and *Al. decemarticulatus* colonies. *Atta* workers were placed on inhabited branches of the respective host tree. The *Atta* ants were then monitored for 30 min and classified into three categories: remained free—F, captured—C or escaped—E. Significant differences between *Az. brevis* and *Al. decemarticulatus* were analysed with a non-parametric Mann–Whitney *U* test. Further details are in the text. **P ≤ 0.005, *P ≤ 0.05, *ns* not significant

When the branches with carton galleries were disturbed, workers of the respective *Az. brevis* colony adopted positions beneath the holes with their mandibles wide open (Fig. 2a). After the first 5 min of the experiment, wherein *Atta* workers were placed on branches with galleries, 77.8% of the individuals were still free, only 18.5% were immediately captured, and 3.7% escaped by simply dropping off the tree. The capture rate increased constantly: 37.0% were captured and immobilised after 10 min; 63.0% after 30 min (Figs. 2c, 3). After 30 min, 3.7% were still free and 33.0% of the *Atta* individuals avoided capture and dropped off the tree (Fig. 3).

In contrast, 90.1% of the 30 *Atta* workers that were placed on the branches of *Al. decemarticulatus*-occupied trees were captured within the first 5 min (Figs. 2d–f, 3). *Al. decemarticulatus* captured the *At. cephalotes* workers significantly quicker than did the *Az. brevis* workers (after 5 min: Mann–Whitney *U, z* = −5.383, *p* = 0.000, *n* = 57), and even after 20 min, the cumulated capture rate was significantly lower for *Az. brevis* than for *Al. decemarticulatus* (Mann–Whitney *U, z* = −2.668, *p* = 0.008, *n* = 57). After 30 min, the capture rate was not significantly different (Mann–Whitney *U, z* = −1.777, *p* = 0.076, *n* = 57), although in the *Allomerus* experiments, more *Atta* individuals were caught (Fig. 3).

The behaviour, which led to captures by both *Az. brevis* and *Al. decemarticulatus*, was similar. If the *Atta* ants inserted one of their legs into one of the gallery’s holes, the individual beneath the hole immediately grasped that leg with its mandibles (Fig. 2). Other workers rapidly approached and grasped the other legs and antennae of the prey. Once immobilised, the insect was killed. The *Azteca* workers very often simply discarded the dead insects, rarely they cut them into pieces and transported parts into the domatia; the rest remained on the carton gallery (Fig. S2, supplementary material). On the contrary, the *Allomerus* workers always transported the prey to the nearest domatium.

## Discussion

Altogether, our results show a structural and functional convergence in the galleries built by these two ant species that belong to different subfamilies. The galleries result from a similar construction process and the slight variations observed in terms of gallery diameter and hole width may be correlated to the size of the workers (supplementary Table 1). The gallery walls of both ant species are strengthened with fungal mycelia of the order Chaetothyriales (Nepel et al. 2014), a group of Ascomycetes which are frequently found in associations between ants and plants (Voglmayr et al. 2011; Vasse et al. 2017). The structural convergence of the galleries is accompanied by a convergence in their function, as both ant species use them to capture and kill other arthropods. *Az. brevis* and *Al. decemarticulatus* workers hide behind the holes and grasp appendices such as the antennae or legs of insects that settle on the gallery, collectively immobilize them, and kill them. Thanks to this ambushing strategy, both ant species are able to capture arthropods whose bodies are much bigger than their own such as the *Atta* workers in our experiment or long-horned grasshoppers and syrphid flies the remains of which are frequently found on the galleries of *Az. brevis* (Fig. S2, supplementary material).

A closer look at the two systems reveals differences. First, the material used in construction differs considerably. While *Al. decemarticulatus* uses plant trichomes as its primary building material and particles of tissue from the inner domatia walls to paste the trichomes together and to favour the growth of the associated fungus (Dejean et al. 2005; Ruiz-González et al. 2011), *Az. brevis* exclusively uses excavated pith, pieces of bark, or epiphyll leaflets. The use of plant trichomes by *Al. decemarticulatus* might be attributed to its high specificity with its densely hirsute host plant, *H. physophora* (Grangier et al. 2009). *Az. brevis* is more generalist and also found elsewhere than on *T. macrophyllum* including on *Licania* sp. (Chrysobalanaceae), *Lonchocarpus* sp. (Fabaceae), *Grias* sp. (Lecythidaceae), *Myriocarpa* sp. (Urticaceae), and *Ocotea nicaraguensis* (Lauraceae) (Longino 2007b; Nepel et al. 2014), none of which bear trichomes as does *Hirtella*. Such a difference in the construction material might partly explain the greater instability of the *Az. brevis* galleries, which were often damaged when the prey struggled, whereas those constructed by *Al. decemarticulatus* always remained intact.

Second, there are behavioural divergences. The first difference could be observed at the beginning of the predation sequence. *Az. brevis* workers did not seem to wait for prey, and only appeared beneath the holes after the branches were disturbed or after an alarm was triggered, whereas *Al. decemarticulatus* constantly guarded them (pers. observation). Moreover, compared to the congeneric species *Al. octoarticulatus, Al. decemarticulatus* invests more time and energy in gallery construction and use to the detriment of leaf patrolling (Orivel et al. 2017). This may explain why, in the experiment with *Atta* workers, *Al. decemarticulatus* caught the *Atta* workers much quicker and more often than did *Az. brevis*. Furthermore, *Az. brevis* was regularly observed to simply discard the dead insects from the branches. This behaviour did not only concern the introduced *Atta* workers but was also observed for other potential prey insects (e.g., mosquitoes, treehoppers, and small lepidopteran larvae) and contrasts with the behaviour of *Al. decemarticulatus*. Unlike *Az. brevis*, which tends “herds” of coccids of the genus *Cryptostigma* (Fig. S1C, supplementary material) that supply the ants not only with carbohydrate-rich exudates, but may also provide protein and lipids if eaten (Gullan 1997; Billick et al. 2007); *Al. decemarticulatus* does not tend coccids and is mainly a predatory species. This difference in the diet of the two ant species might explain the observed variation in prey capture and indicate a different level of specialization in their hunting strategies and thus different functions of the galleries.

Indeed, *Al. decemarticulatus* use the galleries mainly for prey capture. In contrast, *Az. brevis* galleries seem to have mainly a protective function. As the main nest is spread over the entire tree and located in branches bearing a cavity of only a few millimetres in diameter (see Supplementary Fig. S2 B, C), traffic inside this elongated nest (estimated up to 100-m-long if all of the branches are placed end-to-end) may be difficult. The runway galleries allow *Az. brevis* to move quickly and safely without being exposed to enemies and in case of danger to defend the nest efficiently from predators. The use of killed enemies as a food source seems to be a secondary benefit rather than the main function of the *Az. brevis* galleries.

This dual function, prey capture, and defense, emerged from the structure of the galleries, as the holes allow the workers to enter and exit the galleries elsewhere other than just at the extremities. Since both studied ant species are associated with ant-plants, the defensive/predatory roles of the galleries also benefit the plant in terms of biotic protection against herbivores as shown in the experiment with leaf-cutter ants (but see also Orivel et al. 2017).

## Electronic supplementary material

Below is the link to the electronic supplementary material.

Supplementary material 1 (DOCX 825 KB)
