# Supplementary material for: Convergent structure and function of mycelial galleries in two unrelated Neotropical plant-ants
Source: Insectes Soc. 2017 Mar 11;64(3):365–71. doi: 10.1007/s00040-017-0554-y (PMC5509771; doi:10.1007/s00040-017-0554-y)
Supplement: Supplementary file 1 — Supplementary material 1 (DOCX 825 KB) [file 40_2017_554_MOESM1_ESM.docx]

**SUPPLEMENT**

**Supplementary Table 1.** Range of the gallery dimensions and ant morphological traits.

|  |  | ***Azteca brev*is**  (n=5) | ***Allomerus decemarticulatus***  (n=10) |
| --- | --- | --- | --- |
| **Gallery dimensions [mm]** | Inner width | 3.1-4.2 | 2.9-3.0 |
|  | Hole diameter | 0.6-1.6 | 0.7-0.9 |
|  | Hole distance | 1.3-6.0 | 2.0-4.5 |
| **Ant traits [mm]** | Head width | 0.96-1.00 | 0.53-0.60 |
|  | Head length | 1.07-1.09 | 0.63-0.78 |
|  | Total body length | 2.03-3.00 | 1.93-2.51 |


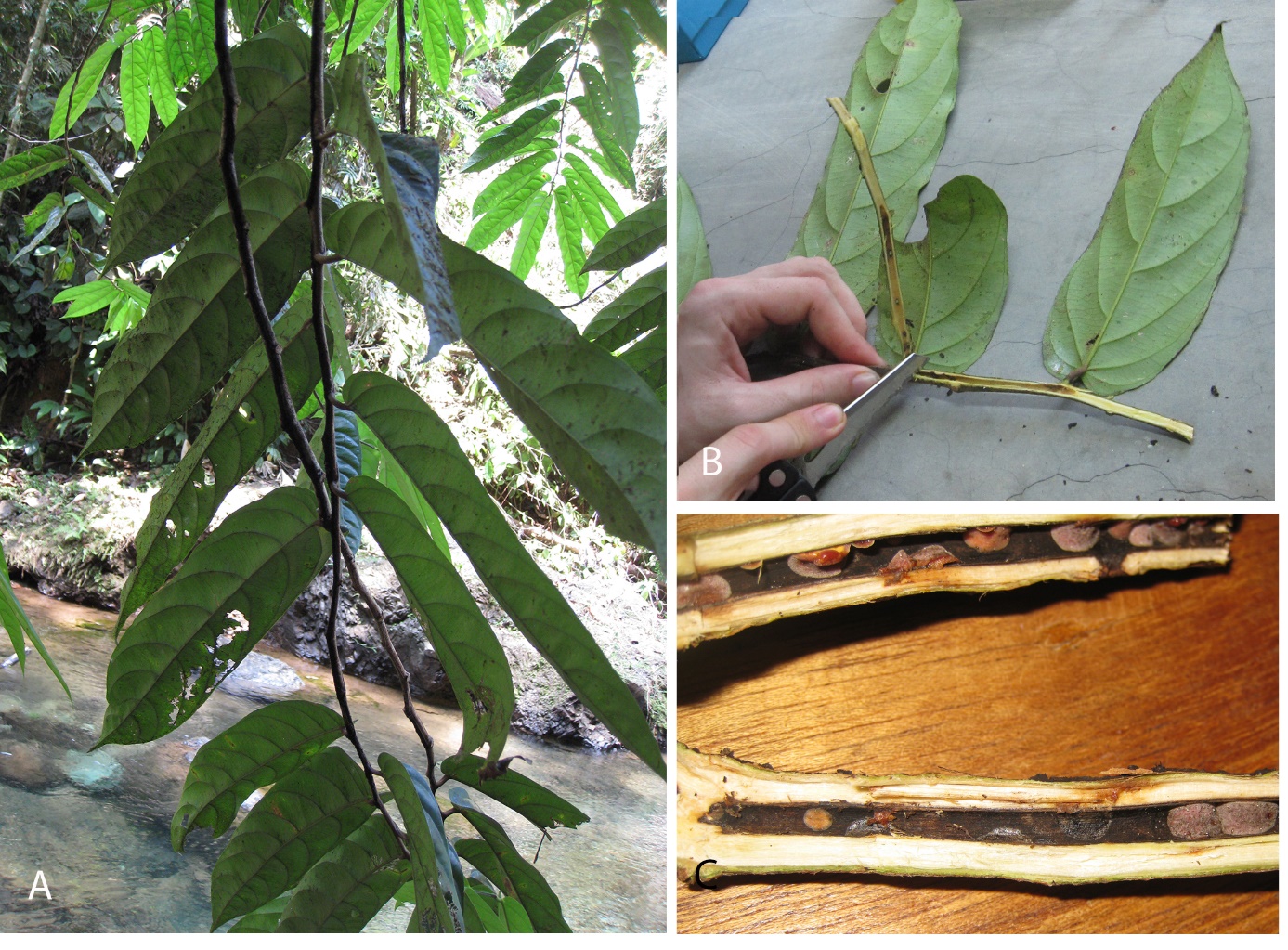


**Supplementary Figure S1.** Branches of *Tetrathylacium macrophyllum* inhabited by *Az. brevis*. **A** Black carton galleries covering the underside of the branches, **B** opening a branch, **C** the cavity is narrow but usually along the length of the whole branch. The branch shown here is full of *Cryptostigma* sp. coccids.


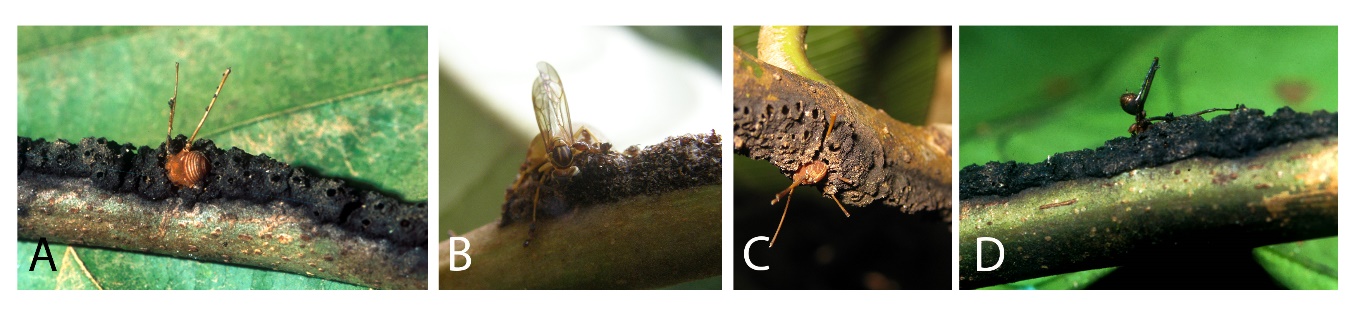


**Supplementary Figure S2.** Different insects captured and killed by *Az. brevis*. They usually simply discard the killed insects, but sometimes they leave them on the carton galleries. **A, C** the remains of grasshopper, **B** of a syrphid fly, **D** of an ant.
